# The effects of menopause on the quality of life and longterm outcomes of transobturator tape treatment in women with stres urinary incontinence

**DOI:** 10.1590/S1677-5538.IBJU.2019.0331

**Published:** 2020-07-31

**Authors:** Mehmet Oguz Sahin, Volkan Sen, Bora Irer, Guner Yildiz

**Affiliations:** 1 Manisa State Hospital Department of Urology Manisa Turkey Department of Urology, Manisa State Hospital, Manisa, Turkey; 2 Izmir Metropolitan Municipality Esrefpasa Hospital Department of Urology Izmir Turkey Department of Urology, Izmir Metropolitan Municipality Esrefpasa Hospital, Izmir, Turkey; 3 Dr. Suat Seren Chest Diseases and Surgery Training and Research Hospital Department of Urology Izmir Turkey Department of Urology, Dr. Suat Seren Chest Diseases and Surgery Training and Research Hospital, Izmir, Turkey

**Keywords:** Menopause, Urinary Incontinence, Stress, Quality of Life

## Abstract

**Purpose:**

We aimed to investigate the effects of menopause on long-term outcomes of transobturator tape (TOT) surgery.

**Materials and Methods:**

Patients who underwent TOT surgery were evaluated under two groups as postmenopausal and premenopausal. The International Consultation on Incontinence short-form questionnaire (ICIQ-SF), Incontinence Impact Questionnaire (IIQ-7) and Urogenital Distress Inventory-Short Form (UDI-6) questionnaires were completed by the patients at the 1^st^ and 5^th^-year follow-up sessions. Patients with a postoperative UDI-6 and IIQ-7 score of <10 were considered as cured, those with lower postoperative scores compared to the preoperative period were regarded as improved, and the cases that had higher postoperative scores than preoperative values were interpreted as TOT failure. The TOT success rates were compared between the results obtained from UDI-6 and IIQ-7.

**Results:**

A total of 109 patients were included in the study (53 postmenopausal and 56 premenopausal). We contacted with 90 (48 premenopausal and 42 postmenopausal) women at 1^st^ year control and 80 (44 premenopausal and 36 postmenopausal) women at 5^th^ year control. There was a significant improvement in all of three questionnaires between the preoperative and post-operative 1^st^ year control (ICIQ-SF: 15.5±2.5 vs. 1.8±4.3, p <0.001; IIQ-7: 68.9±9.8 vs. 2.75±15.2, p <0.001; UDI-6: 27.1±11.1 vs. 6.0±14.6, p <0.001) and the preoperative and post-operative 5^th^ year control (ICIQ-SF: 15.5±2.5 vs. 3.1±5.3, p <0.001; IIQ-7: 68.9±9.8 vs. 9.6±26.7, p <0.001; UDI-6: 27.1±11.1 vs. 5.1±10.0, p <0.001). When we compared the premenopausal and postmenopausal patients in terms of recurrent urinary tract infection (UTI); 5 (12%) patients had recurrent UTI in postmenopausal group but no patients had recurrent UTI in premenopausal group at 1^st^ year follow-up (p=0.039) and similarly the same 5 (13.9%) patients in follow-up had recurrent UTI in postmenopausal group but no patients had recurrent UTI in premenopausal group at 5^th^ year follow-up (p=0.045). There were no significant differences between the premenopausal and postmenopausal patients in terms of TOT success rates at 1^st^ and 5^th^ year control, evaluated with UDI-6 (1st year: p=0.198 and 5^th^ year: p=0.687) and IIQ-7 (1^st^ year: p=0.489 and 5^th^ year: p=0.608) questionnaires.

**Conclusions:**

Transobturator tape surgery is an effective and reliable method according to the long-term outcomes reported in this paper. In the current study, we determined that the TOT success rates were not affected by the presence of menopause.

## INTRODUCTION

Urinary incontinence (UI) has been defined as involuntary urine loss by the International Continence Society (ICS) (1). Risk factors for UI include age, obesity and excess adipose tissue, parity, pregnancy, hormone replacement therapy for menopause, ethnicity and race, hysterectomy, dietary factors, socioeconomic status, smoking, physical activity, and comorbidities (diabetes, urinary tract infection (UTI), cognitive disorder, ischemic heart disease, physical disorders, and depression) (1, 2). Stress urinary incontinence (SUI), one of the most common types of UI, occurs when the bladder pressure exceeds urethral resistance due to increased abdominal pressure in exercise, sneezing, or coughing, and its prevalence ranges from 4 to 35% in the literature (3, 4). Transobturator tape (TOT) treatment was first described in 2001 and presents as an alternative to the mid-urethral sling technique, in which the synthetic mesh is inserted through the obturator foramen (5).

With menopause and advancing age, all urogynecologic disorders have been shown to increase. Menopause results in the reduction of the maximal urethral closure pressure. At the same time, bladder capacity and detrusor pressure during voiding is significantly decreased in the elderly population (6). On the other hand, estrogen receptors have been identified along the pelvic floor trigone and uterosacral ligaments including the urethra, vagina and bladder, and estrogen loss was often associated with urogenital atrophy and urinary symptoms after menopause (7). Stress urinary incontinence is more commonly seen among postmenopausal patients, and studies in the literature have reported different results in term of the success and complication rates of TOT surgery in premenopausal and postmenopausal patients (8, 9).

In this study, we aimed to investigate the effects of menopause on the long-term outcomes of the TOT operation, which is defined as a minimally invasive technique.

## MATERIALS AND METHODS

After obtaining the approval of the local ethics committee, patients who underwent suburethral vaginal TOT surgery due to SUI in our urology clinic between January 2008 and June 2013 were retrospectively reviewed. Patients were evaluated under two groups as postmenopausal and premenopausal before the TOT operation. Life style changes including weight loss and pelvic muscle exercises were offered before and after the TOT surgery. Demographic characteristics of the patients, International Consultation on Incontinence short-form questionnaire (ICIQ-SF), Urogenital Distress Inventory-Short Form (UDI-6) and Incontinence Impact Questionnaire (IIQ-7) questionnaire results, examination findings, stress test (MMK: Marshall-Marchetti-Krantz), Q-tip test, operation results, and complications were noted. Patients who previously had surgery for UI or pelvic organ prolapse, those having a marked neurological disease, urgency urinary incontinence (UUI), or cystocele or rectocele at the degree that would require surgical repair, and those who were using medication that made them prone to bleeding and the patients who were premenopausal before the surgery and then became menopausal during the time interval of study were excluded from the study. None of the patients with menopause took any hormonal therapy in their follow-up. TOT operation decision was scheduled for patients that had no preoperative pathology according to the urodynamic testing, a Q-tip test result of >30 degrees, and a positive stress test result. All operations were performed using the outside-in method under spinal anesthesia. Polypropylene mesh (Uni-tape T-Promedon®) was used as the TOT material during the operation. Cystoscopy was not routinely performed to all patients.

Urogynecologic examinations were performed on the patients. Q-tip angle, MMK test, operation success, and per-operative complications were recorded. The Turkish versions of the ICIQ-SF, IIQ-7 and UDI-6 questionnaires, which had been previously validated, were completed by the patients again at the first- and fifth-year follow-up sessions (10, 11). The outcomes of the operation were classified according to the subjective evaluations of the patients through the questionnaires: Patients with a postoperative UDI-6 and IIQ-7 score of <10 were considered as cured, those with lower postoperative scores compared to the preoperative period were regarded as improved, and the cases that had higher postoperative scores than preoperative values were interpreted as TOT failure (12). The overall success rate of TOT was defined as sum of the cure and improvement rates. TOT success rates were compared between the groups at 1^st^ and 5^th^ year outcomes.

Data were analyzed using the Statistical Package for Social Sciences (SPSS, Inc., Chicago IL) version 22 and expressed as mean±standard deviation, number (n) and percentage (%) values. In the comparison of categorical variables between the groups, the X^2^ test and Student’s t-test were employed to compare continuous variables. A p value of 0.05 was considered to be statistically significant.

## RESULTS

**Figure 1:**
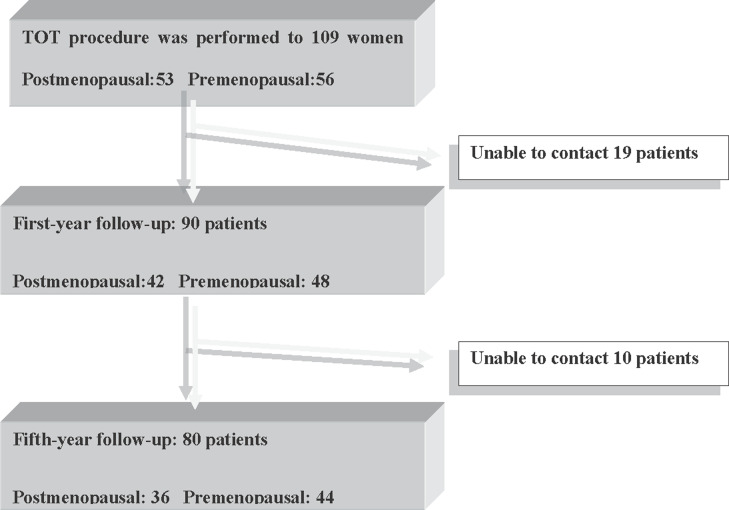
The first-year and fifth-year distribution of the patients that underwent TOT surgery.

A total of 109 patients were included in the study and their mean age was 52.4±10.1 (2776) years. There were 53 postmenopausal (48.6%) and 56 premenopausal (51.4%) women. The mean body mass index (BMI) of the patients was 26.2±4.0 (20.2-34.4) kg/m^2^. The mean Q-tip test angle was 57.0±17.1 (30-90) degrees and the mean follow-up period was 74.4±46.2 (1–138) months. The mean operation time was 29.7±9.7 (12-60) minutes, and the mean hospitalization time was 1.2±0.5 (1-3) days. For all patients, the cough (MMK) test was positive and UUI was negative. We contacted with 90 (48 premenopausal and 42 postmenopausal) women at 1^st^ year control and 80 (44 premenopausal and 36 postmenopausal) women at 5^th^ year control (Figure-1).

The overall success rate of TOT procedure was detected as 93.3% at 1^st^ year follow-up and 88.8% at 5^th^ year follow-up according to both IIQ-7 and UDI-6 questionnaires. There was no significant difference between the success rates of premenopausal and postmenopausal patients at 1^st^ year follow-up (93.7% vs. 92.9%, p=0.865) and 5^th^ year follow-up (90.9% vs. 86.1%, p=0.499) (Table-1). The cure rates were also similar between the premenopausal and postmenopausal patients according to IIQ-7 (83.3% vs. 78.6%, p=0.821) and UDI-6 (70.8% vs. 54.8%, p=0.648) at 1^st^ year; IIQ-7 (75.0% vs. 69.4%, p=0.485) and UDI-6 (75.0% vs. 69.4%, p=0.485) at 5^th^ year follow-up (Table-1).

**Table 1:** Comparison of the cure and success rates of postmenopausal and premenopausal groups in terms of the first-year and fifth-year outcomes according to IIQ7 and UDI-6.

	Postoperative 1st year (90 patients; 48 premenopausal, 42 postmenopausal)						Postoperative 5th year (80 patients; 44 premenopausal, 36 postmenopausal)					
IIQ-7	Failure	Cure	P*	Improvement	Success	P**	Failure	Cure	P*	Improvement	Success	P**
	6 (6.7%)	73 (81.1%)		11 (12.2%)	84 (93.3%)		9 (11.2%)	58 (72.5%)		13 (16.3%)	71	
											(88.8%)	
**Postmenopausal**	3 (7.1%)	33 (78.6%)		6 (14.3%)	39 (92.9%)		5 (13.9%)	25 (69.4%)		6 (16.7%)	31	
											(86.1%)	
			0.821			0.865			0.485			0.499
**Premenopausal**	3 (6.3%)	40 (83.3%)		5 (10.4%)	45 (93.7%)		4 (9.1%)	33 (75.0%)		7 (15.9%)	40	
											(90.9%)	
	Failure	Cure	P*	Improvement	Success	P**	Failure	Cure	P*	Improvement	Success	P**
**UDI-6**	6 (6.7%)	57 (63.3%)		27 (30.0%)	84 (93.3%)		9 (11.2%)	58 (72.5%)		13 (16.3%)	71	
											(88.8%)	
**Postmenopausal**	3 (7.1%)	23 (54.8%)		16 (38.1%)	39 (92.9%)		5 (55.6%)	25 (69.4%)		6 (16.7%)	31	
											(86.1%)	
			0.648			0.865			0.485			0.499
**Premenopausal**	3 (6.3%)	34 (70.8%)		11 (22.9%)	45 (93.7%)		4 (44.4%)	33 (75.0%)		7 (15.9%)	40	
											(90.9%)	

**IIQ-7** = Incontinence Impact Questionnaire; **UDI-6**: Urogenital Distress Inventory-Short Form.

* = Chi-square test; p values show the comparison of the premenopausal and postmenopausal patients according to failure and cure rates.

** = Chi-square test; p values show the comparison of the premenopausal and postmenopausal patients according to failure and success rates.

When we compared the premenopausal and postmenopausal patients in terms of recurrent urinary tract infection (UTI); 5 (12%) patients had recurrent UTI in postmenopausal group but no patients had recurrent UTI in premenopausal group at 1^st^ year follow-up (p=0.039) and similarly the same 5 (13.9%) patients on follow-up had recurrent UTI in postmenopausal group but no patients had recurrent UTI in premenopausal group at 5^th^ year follow-up (p=0.045). No new recurrent UTI was added between 1^st^ and 5^th^ year follow-up. While 22 (20.2%) women had urgency in the preoperative period, the postoperative new urgency was observed in three patients (3.3%) in 1^st^ year and 11 patients (13.8%) in 5^th^ year. In addition, no UUI was found in the preoperative period whereas UUI was observed to newly develop in five patients (5.6%) in the first-year and 11 (13.8%) patients in the fifth-year follow-up.

There was a significant improvement in all of the three questionnaires between the preoperative and post-operative 1^st^ year control (ICIQ-SF: 15.5±2.5 vs. 1.8±4.3, p <0.001; IIQ-7: 68.9±9.8 vs. 2.75±15.2, p <0.001; UDI-6: 27.1±11.1 vs. 6.0±14.6, p <0.001) and the preoperative and post-operative 5^th^ year control (ICIQ-SF: 15.5±2.5 vs. 3.1±5.3, p <0.001; IIQ-7: 68.9±9.8 vs. 9.6±26.7, p <0.001; UDI-6: 27.1±11.1 vs. 5.1±10.0, p <0.001). The preoperative Q-tip test angles were significantly lower in the postoperative 1^st^ and 5^th^ years (p <0.001), which is an indication of the efficacy and permanence of the positive outcomes of the operation (Table-2).

**Table 2:** The comparison of the questionnaires scores, UI symptoms and physical examination results of patients according the follow - up years (preoperative vs postoperative 1^st^ year and preoperative vs postoperative 5^th^ year).

	Preoperative (n=109)	Postoperative 1^st^ year (n=90)	Postoperative 5^th^ year (n=80)	P*
MMK test	0 (0%)	85 (94.4%)	73 (91.3%)	<0.001
Q tip (°)	57.0±17.1	17.1±12.5	19.8±15.9	<0.001
SUI symptoms	109 (100%)	6 (6.7%)	9 (11,3%)	<0.001
De-novo UUI	-	5 (5.6%)	11 (13.8%)	-
ICIQ-SF	15.5±2.5	1.8±4.3	3.1±5.3	<0.001
IIQ-7	68.9±9.8	2.75±15.2	9.6±26.7	<0.001
UDI-6	27.1±11.1	6.0±14.6	5.1±10.0	<0.001

**MMK** = Marshall-Marchetti-Krantz; **SUI** = Stress urinary incontinence; **UUI** = Urge urinary incontinence; **ICIQ-SF** = International Consultation on Incontinence short-form questionnaire; **IIQ-7** = Incontinence Impact Questionnaire; **UDI-6** = Urogenital Distress Inventory-Short Form.

* = p values show both of the comparison of preoperative vs postoperative 1st year and preoperative vs postoperative 5th year outcomes.

There was no significant difference between the postmenopausal and premenopausal patients in terms of BMI, Q-tip angle, follow-up duration, operation time, and hospitalization time according to the evaluations undertaken in the 1^st^ and 5^th^ years (p >0.05) (Table-3). Three postmenopausal and two premenopausal women had a positive result in the cough test in the 1^st^ year, with no statistically significant difference (p=0.564). At 5^th^ year of follow-up, premenopausal group had 3 patients with positive cough test, while post-menopausal group had four. The difference was not significant (p=0.419). Concerning systemic diseases, there was no difference between the 1^st^ year and 5^th^ year results of the groups (p=0.734 and p=0.838, respectively) (Table-3).

**Table 3:** Comparison of the demographic characteristics, surgical outcomes and complications of the postmenopausal and premenopausal groups in terms of follow-up years.

		Postoperative 1.st year (n=90)			Postoperative 5.th year (n=80)		
		Premenopausal n=48 (53.3%)	Postmenopausal n=42 (46.7%)	P	Premenopausal n=44 (55%)	Postmenopausal n=36 (45%)	P
BMI (kg/m^2^)		27.1±4.2	26.6±3.6	0.370	27.2±4.2	26.3±3.6	0.234
Follow-up time (months)		94.8±33.1	83.6±37.7	0.148	102.3±22.5	95.5±25.2	0.210
Operation time (min)		29.7±10.6	29.5±9.0	0.931	29.5±10.8	29.6±9.7	0.978
Hospitalization (days)		1.2±0.6	1.1±0.3	0.264	1.3±0.6	1.1±0.3	0.200
**Co-morbidities**							**0.838**
	No	26 (54.2%)	27 (64.3%)	0.734	24 (54.5%)	22 (61.1%)	
	Diabetes Mellitus	8 (16.7%)	5 (11.9%)		7 (15.9%)	5 (13.9%)	
	Hypertension	5 (10.4%)	4 (9.5%)		5 (11.4%)	4 (11.1%)	
	Thyroid diseases	3 (6.3%)	3 (7.1%)		2 (4.5%)	3 (8.3%)	
	COPD	4 (8.3%)	1 (2.4%)		4 (9.1%)	1 (2.8%)	
**Complications**							**0.172**
	No	42 (87.5%)	40 (95.2%)	0.156	40 (90.1%)	34 (94.4%)	
	Bleeding	4 (8.3%)	0 (0.0%)		3 (6.8%)	0 (0.0%)	
	Acute urinary retention	2 (4.2%)	0 (0.0%)		1 (2.3%)	0 (0.0%)	
	Bladder injury	0 (0.0%)	2 (4.8%)		0 (0.0%)	2 (5.6%)	
MMK(+)		2 (4.2%)	3 (7.1%)	0.564	4 (9.1%)	3 (8.3%)	0.419
Q tip (°)		16.4±13.0	17.9±11.9	0.571	19.7±16.1	19.9±16.0	0.955
SUI (+)		3 (6.3%)	3 (7.1%)	0.542	4 (9.1%)	5 (13.9%)	0.319
De-novo urgency		2 (4.2%)	1 (2.4%)	0.550	5 (11.4%)	6 (16.7%)	0.344
De-novo urge incontinence		3 (6.3%)	2 (4.8%)	0.564	5 (11.4%)	6 (16.7%)	0.332
Recurrent UTI		0 (0.0%)	5 (12.0%)	0.039	0 (0.0%)	5 (13.9%)	0.045
Dyspareunia		6 (12.5%)	3 (7.1%)	0.314	5 (11.4%)	2 (5.6%)	0.307
UDI-6		7.1±13.0	10.7±18.1	0.274	9.8±16.8	12.8±19.8	0.453
IIQ-7		7.6±18.7	10.9±20.3	0.421	15.1±30.3	17.5±24.9	0.699
ICIQ-SF		1.8±4.6	1.8±4.0	0.984	2.9±5.9	3.3±4.8	0.776

**BMI** = Body mass index; **DM** = Diabetes Mellitus; **HT**: Hypertension; **COPD** = Chronic obstructive pulmonary disease; **MMK** = Marshall-Marchetti-Krantz; **SUI** = Stress urinary incontinence; **UTI**: Urinary tract infection; **UDI-6** = Urogenital Distress Inventory-Short Form; **IIQ-7** = Incontinence Impact Questionnaire; ICIQ-SF = International Consultation on Incontinence short-form questionnaire.

There was no significant difference between the postmenopausal and premenopausal groups in terms of the 1^st^ and 5^th^ year complication rates (p=0.156 and p=0.172, respectively). In the peroperative period, four women (3.7%) had bleeding that resulted in a blood loss of greater than 200mL and only one (0.9%) required erythrocyte suspension transfusion. All four patients were premenopausal. Bladder injuries were found in two (1.8%) women; thus, for these cases, the catheter was withdrawn seven days later and the treatment was initiated. Both these patients were postmenopausal. Except for two women with bladder injuries, all catheters were withdrawn on the first postoperative day.

In the early postoperative period, two patients had acute urinary retention (AUR), and kept the urinary catheter for 7 days. They had no other issues during follow-up. Both women that developed AUR were in the premenopausal group. Among the late postoperative complications evaluated, there was no difference between the post menopausal and premenopausal groups in terms of de novo urgency, de novo UUI, dyspareunia, perineal pain, and vaginal discharge in 1^st^ and 5^th^ year follow-ups (p >0.05) (Table-3).

## DISCUSSION

Urinary incontinence constitutes an important sociocultural health problem with high prevalence. Considering that the quality of life has attained more importance and the average life expectancy has extended, SUI treatment will become more valuable in the coming years with the increased number of geriatric individuals.

Studies showing the effects of menopause on various surgical techniques applied in the treatment of SUI report different results. While some emphasize the negative effects of menopause, others suggest that there is no such effect. In 2010, Rechberger et al. concluded that both menopause and aging had a detrimental effect on the ultimate outcome of both retropubic and transobturator sling due to the development of textural and hormonal changes (13). The study undertaken by Polat et al. confirms this finding, they found no significant difference in the mean operation time, length of hospital stay or intraoperative and postoperative complications, but noted that the premenopausal women were more satisfied with the surgery than the postmenopausal women. In addition, the improvement in the UDI-6 scores of premenopausal women was more significant (8). In contrast to these results, researchers investigating the effect of menopause on the success and failure rate of the Burch procedure examined 258 patients and observed no effect of menopause on the failure rate (14). Yasa et al. showed that TOT surgery in SUI treatment had high rates of success and patient satisfaction and a low postoperative morbidity rate in postmenopausal women aged over 65 years (15). Agarwal et al. reported that TOT surgery was more successful in premenopausal women younger than 50 years with a urethral mobility greater than 30 degrees (16). In the present study, there was no difference between the postmenopausal and premenopausal groups in terms of TOT success and fifth-year follow-up evaluations. We did not find any difference in the duration of follow-up, operation time, and hospitalization time between the two groups. According to the results of the UDI-6 and IIQ-7 questionnaires, the postmenopausal and premenopausal groups did not differ in relation to the rates of TOT failure, TOT success and clinical improvement.

Arrabal-Polo et al. calculated the total complication rate of TOT surgery as 12% in their study (17). Kaelin-Gambirasio et al. found the peroperative and early postoperative complication rate to be 9.5% (18). In our study, the peroperative and early postoperative complication rate being 7.2% confirms the safety of the TOT operation.

Richter et al. comparatively evaluated patients under 65 years (34.6% premenopausal) and over 65 years (all postmenopausal) that underwent the Burch colposuspension or pubovaginal sling for the treatment of SUI and found no difference in the complication rates (including hemorrhage) of the two groups (19). Yasa et al. did not observe any bladder perforation among the postmenopausal patients aged below and over 65 years that underwent TOT surgery due to SUI and noted no statistically significant difference between the groups concerning other complications observed, namely voiding dysfunction, vaginal erosion, de novo urgency, and suprapubic or hip pain (15). In the current study, considering the four patients with a blood loss of more than 200mL, the rate of hemorrhage was 2.8%.

Voiding difficulty is another complication that develops in the postoperative period. Urinary obstruction that occurs in the first few days may be due to edema and pain, but in the following days, specifically within 10 days, this effect is minimized and the patient is expected to perform the voiding function without difficulty (20). In the present study, transient urinary retention developed in two patients (1.8%) in the early postoperative period, and therefore the catheter remained for one week. The patients were able to spontaneously urinate after the catheter was removed. The four patients with hemorrhage and the two patients with acute urinary retention were in the premenopausal group. Bladder injury was seen in only two patients, both in the postmenopausal group. Despite the absence of a statistically significant difference between the two groups in terms of complications, the presence of hemorrhage in four and acute urinary obstruction in two premenopausal patients could be explained by congestion, blood supply and edema due to estrogen, and similarly, the two cases of bladder injury being seen in the postmenopausal group can be attributed to the reduced level of estrogen, resulting in tissue thinning and increased fragility.

Another condition that can be seen after TOT operations is de novo urgency incontinence, which was identified at a rate of 5.6% in the first year and 13.8% in the fifth year of the current study. Consistent with the report of Yasa et al., we found no difference between the postmenopausal and premenopausal groups (15).

Urinary tract infection, another complication encountered after mid-urethral sling operations, occurs at a rate of 34% in the first three months and 50% in the first year (21, 22). The prevalence of recurrent UTI is reported as 6.4% in various case series (23). In a study by Weintraub et al., the rate of UTI was found to be 21.3% after mid-urethral sling surgery. When the groups with and without infection were considered, the mean age of the infection group was greater, but without statistical significance. The menopause status was not different in these groups. The authors suggested that UTI had a linear relationship with the presence of perineal hematoma and prolonged hospitalization time (24). In our patient groups, the rate of recurrent UTI was 5.6% in the first year and 6.3% in the fifth-year follow-up. All the patients presenting with recurrent UTI were in the postmenopausal group, and there was a statistically significant difference for the first-year and fifth-year values. As expected, the mean age of the postmenopausal group was higher than that of the premenopausal group. None of the patients with recurrent UTI had peroperative complications. We consider that the reason for detecting increased recurrent UTI in the postmenopausal group is the reduced estrogen, blood supply, and weakening of the bladder mucosal barrier.

The main limitation of this study is its retrospective design. Data about menopause status of patients was only evaluated before TOT surgery also there was no data which patients used hormonal therapy for menopause.

## CONCLUSIONS

Transobturator tape surgery is an effective and reliable method according to the long-term outcomes reported in this paper. In the current study, we determined that the TOT success rates were not affected by the presence of menopause.
